# Estimated Burden of Serious Fungal Infections in Malawi

**DOI:** 10.3390/jof4020061

**Published:** 2018-05-21

**Authors:** Khumbo Kalua, Boston Zimba, David W. Denning

**Affiliations:** 1 Blantyre Institute for Community Outreach (BICO), Blantyre, Malawi; 2 College of Medicine, University of Malawi, Blantyre, Malawi; bzimba@gmail.com; 3 National Aspergillosis Centre, Wythenshawe Hospital and The University of Manchester, Manchester M13 9PL, UK; ddenning@manchester.ac.uk; 4 Leading International Fungal Education (LIFE) (www.LIFE-Worldwide.org), Cheshire SK10 9AR, UK

**Keywords:** fungal, incidence, prevalence, Malawi, HIV/AIDS, epidemiology

## Abstract

Despite efforts to address the burden of fungal infections in Malawi, the prevalence and incidence remain largely unknown. We assessed the annual burden in the general population and among populations at high risk and fungal infection frequencies in each particular population to estimate the national incidence or prevalence. The Malawi population is approximately 17.7 million (2017), with 48% under 15 years of age. Approximately 8% of the population is HIV positive. The most common infections are present in HIV/AIDS patients, with oral candidiasis being the commonest. Life threatening infections among those with AIDS patients include cryptococcal meningitis (8200 cases) and *Pneumocystis* pneumonia (3690 cases). Pulmonary TB is common, but extra pulmonary TB is rare; an estimated 2329 people have chronic pulmonary aspergillosis after TB. Asthma is a significant problem in Malawi, with an estimated 680,000 adults affected (4.67%) and 14,010 cases of allergic bronchopulmonary aspergillosis (ABPA). Tinea capitis is estimated to be present in over 670,000 young people (21% of school age children). The annual incidence of fungal keratitis is difficult to estimate, but as cases are frequently seen in the eye department, is likely to be a minimum of 1825 (10.3/100,000) cases. Among the most serious infections, cryptococcal meningitis and *Pneumocystis* pneumonia are top of the list. Overall, some 1,338,523 (7.54%) people are affected by a serious fungal infection in Malawi. These basic estimates are limited, due to poor record keeping, and require epidemiological studies to validate or modify the substantial burden estimates. National surveillance of fungal infections is urgently needed.

## 1. Introduction

It is estimated that globally, approximately 300 million people suffer from serious fungal infections annually, and that among these, 1.65 million die [1]. As in many poorly resourced African countries, Malawi has no comprehensive data on the burden of fungal infections. The HIV epidemic in Malawi has highlighted *Cryptococcus* and *Candida* infections as important opportunistic fungal infections, and has some data for this, but national data for other fungal infections is not readily available. This is partly because a large number of healthy persons with mild superficial or even severe infections may not present at a health facility, such that the sparse data on life-threatening invasive diseases in vulnerable immunocompromised patients usually underestimates the burden of the disease by a large degree. The low figures reported have often resulted in the conclusion that such fungal infections are not of public health importance, probably making fungal infections one of the most neglected fatal diseases among many neglected diseases. There has been some progress in placing several antifungals on the World Health Organisation Essential Drug List (fluconazole, amphotericin, flucytosine, itraconazole, voriconazole, and topical natamycin) [2]. However, despite serious fungal infections being reported at all major hospitals in Malawi, major broad spectrum antifungals are still not included on the essential drug list in Malawi, and are only available as specially imported drugs for specific diseases. In this paper, we present a review of the epidemiology of fungal disease in Malawi, with estimates based on available data as of 2017.

## 2. Materials and Methods

Reported data from multiple sources including the National statistical office (NSO) were used for compiling the population demographics. This was followed by a thorough PubMed search for fungal disease using the terms; Fungal, fungal infection, fungal epidemiology, fungal burden, HIV, Aids, Malawi, National AIDS commission, keratitis, Africa, and variations thereof. A second search included the same searches using the following diseases; *Cryptococcus*/cryptococcal, *Candida*/thrush, *Aspergillus*/aspergillosis, histoplasmosis, asthma, leukaemia, chronic obstructive pulmonary disease (COPD), *Pneumocystis* pneumonia/*Pneumocystis jirovecii* pneumonia (PJP)/*Pneumocystis carinii* pneumonia, chronic pulmonary aspergillosis (CPA), aspergilloma, allergic bronchopulmonary aspergillosis (ABPA), severe asthma with fungal sensitisation (SAFS), and tinea/ringworm. National or local information on these conditions was used wherever available, and if not available, data were used in the following preferential order; Sub-Saharan Africa, East Africa, rest of the world. Where no available data were found in the literature, authors were sought for local unpublished data and government personnel working in the health sector, or other departments, were contacted for the provision of any information that was available. Where we could not get any local data, we applied estimates from neighbouring countries/regions. It should be noted that we have only used a narrative approach in reporting, as it was not possible to use conventional comparative methods due to heterogeneity of the data. As a result, our estimates are based on the lowest incidence rates that were reported in well-defined populations.

## 3. Results and Discussion

### 3.1. Country Profile

Malawi is a very densely populated country, with approximately 18 million people (current fertility is estimated at 4.4 children per woman) [3]. Youth younger than 15 account for 46–48% of the population. The gross domestic product per capita was $301 in 2016. HIV prevalence among adults ages 15 to 64 years is 10.6%, with annual incidence of the same group at 0.37% (28,000 new cases of HIV annually). HIV prevalence among children is 1.6%. There are an estimated 979,896 people living with HIV (PLHIV) in Malawi, about 104,093 of whom are children younger than 15 years. HIV prevalence among 15–49 year-olds declined from a peak of 16.7% in 1999 to 10.0% in 2016. Annual HIV incidence declined from a peak of 110,000/year in 1998 to 85,000/year in 2004, before dropping to an estimated 28,000/year in 2016. HIV prevalence remains disproportionately higher among females than males; HIV prevalence is three times higher among 25–29 year old females than males, pointing to a higher HIV incidence among females than males aged 15–24, for a number of reasons, including girls being more vulnerable—due to being coerced by older men. The Joint United Nations Programme on HIV/AIDS (UNAIDS) estimates that 66% are receiving antiretroviral therapy. There were an estimated 24,000 deaths from AIDS in 2016 (UNAIDS), a significant fall since the peak in 2004.

### 3.2. Epidemiology of Fungal Infections

Among these, the most common infection present in HIV/AIDS patients is oral candidiasis (approximately 216,000 cases) (Table 1). We have assumed that 90% of patients with low CD4 counts develop oral candidiasis at some time over the following two years. Cross-sectional surveys find lower rates, but very few longitudinal studies have been conducted [4]. Oesophageal candidiasis is also common, and often undiagnosed. We estimate that 73,000 cases occur in HIV patients in Malawi each year, based on a 20% rate among those with low CD4 counts not on antiretroviral therapy (ART) and a 5% rate of those on ART [5].

Common life threatening infections among the HIV/AIDS patients include cryptococcal meningitis (with 10% of AIDS patients presenting with the disease) and *Pneumocystis* pneumonia (PCP) (9% of AIDS patient presenting with this). Our estimate of cryptococcal meningitis is substantially higher than a recent global and country estimate because of the basis for estimation [6]. We have based our estimate on numbers of patients presenting with AIDS (with the assumptions above), whereas Rajasingham et al. have based their estimate (3135 cases annually) on cryptococcal antigen prevalence among those with CD4 counts <100 × 10^6^/mL (1.9%). There may be regional differences within the country. The *Pneumocystis* pneumonia (PCP) estimate of 9% is derived from Hargreaves et al., who investigated the causes of disease in adults with smear negative TB [7], using bronchoscopy and both immunofluorescence and PCR. In fact, other estimates are 11% [8] in adults in the community using both immunofluorescence and PCR on induced sputum, and 5% in HIV-positive children using lung aspiration and PCR [9].

There are an estimated 27,000 deaths from AIDS in Malawi each year. Assuming that 4% of these patients have invasive aspergillosis (IA) [10], 1080 patients die of this infection each year, as there are currently no realistic means of making the diagnosis.

Pulmonary TB is present in approximately 11,000 people per year, but extrapulmonary TB is rare. A substantial proportion are ‘smear negative’ [7] or clinically diagnosed, although some of these are later culture confirmed (17%). Updated data on such cases with GeneXpert are not available; the WHO estimate from 2015 shows that overall, 59% were bacteriologically confirmed. It is likely that many of these cases in HIV positive and negative patients are attributable to fungi, including *Aspergillus* spp and *Histoplasma capsulatum*. Three cases of histoplasmosis are described from Malawi, two of which are in HIV positive patients. One patient with disseminated histoplasmosis in AIDS has been described from Malawi [11], so it is likely that histoplasmosis is substantially underdiagnosed.

Chronic pulmonary aspergillosis (CPA) may mimic TB (although fever is unusual in CPA), with weight loss, fatigue, cough, dyspnoea, breathlessness, and haemoptysis. No studies estimating the annual incidence or prevalence of CPA have been conducted in Malawi. A cross sectional study in Nigeria [12] found an overall rate of 8.7%, with a slightly higher proportion in non-HIV patients of 14.5%, with the highest rate in smear negative non-HIV patients (19%). We have used the prior published model of CPA [13] (22% of patients with cavities after TB and 2% of those without cavities after TB) to estimate the annual incidence (730 people and five-year period prevalence of CPA after TB (2329 people), with the latter assuming a 15% annual mortality. CPA affects patients with a wide cross-section of patients with other pulmonary diseases, so we assumed that TB was the underlying diagnosis in 80% (giving an overall prevalence of 16.4/100,000).

Asthma is a significant problem in Malawi, with an estimated rate in adults of 4.67% [14], which translates to 680,000 affected. We have applied a 2.5% rate to estimate the prevalence of ABPA, based on referral to secondary care in South Africa [15], a prevalence estimate of 11,190. While some patients with ABPA have severe asthma, many do not and fungal sensitisation in asthma is strongly associated with severe asthma, especially in adulthood. We also therefore assumed that 10% of the adult asthmatic population have severe asthma and that a conservative proportion of 33% are sensitised to fungi. Such patients—severe asthma with fungal sensitization—may number as many as 14,771 in Malawi, and many would respond to antifungal therapy [16]. There are no data from Africa (except for South Africa) on fungal sensitisation, either the frequency or genus of fungus associated with sensitisation.

Invasive fungal infections in the hospital setting (non-HIV) are usually caused by *Candida* spp. or *Aspergillus* spp. Blood cultures are infrequently drawn in Malawi, so the incidence of candidaemia and invasive candidiasis is not known. It could be low because fewer intravenous catheters are used, there is little renal dialysis, diabetes is less prevalent, and fewer patients are subjected to modern medical management in intensive care and complex chemotherapy and other immunosuppressive therapies. On the other hand, antibiotics are freely used and infection control procedures are often lacking. In the absence of data, we have estimated a candidaemia incidence of 5/100,000 (750 patients). Ordinarily, the invasive candidiasis rate would be over two times as high, partly because of the insensitivity of blood culture, but also because of intra-abdominal candidiasis complicating intestinal perforation and laparotomy and chronic ambulatory peritoneal dialysis.

In addition to Invasive aspergillosis (IA) complicating HIV infection, this difficult-to-diagnose entity complicates leukaemia, transplantation, lung cancer, chronic obstructive pulmonary disease (COPD) (especially when admitted to hospital with an exacerbation), liver failure, intensive care, and corticosteroid therapy, as well as other patients receiving immunosuppressive therapy. We have assumed that 10% of those with acute myeloid leukaemia develop IA and an equal number of IA cases occur in all other haematological conditions in Malawi. There is no organ transplantation done in Malawi. IA also complicates lung cancer, but a reliable estimate in Malawi is not possible, as lung cancer incidence is poorly documented and treatment options are limited. Likewise, prevalence and incidence data for all other underlying conditions in Malawi are not collected, so no reliable estimate is possible. Overall, the annual incidence of IA is at least 1186 (6.7/100,000).

**Figure 1:**
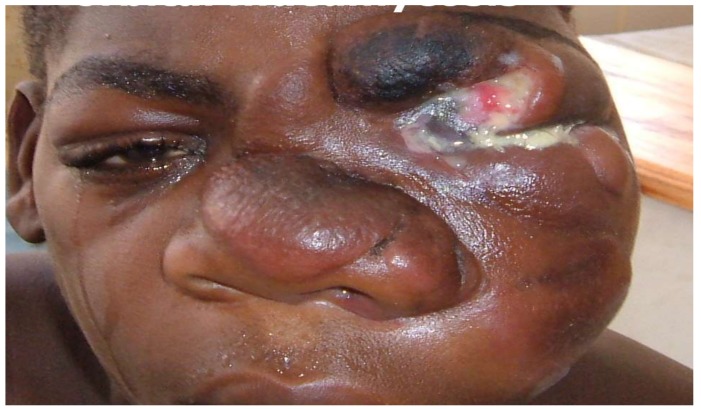
An eye patient presenting at Lions sight first eye hospital in Blantyre Malawi, with a fungal infection (conidiobolomycosis) which has distorted the eye and the overall face.

Mucormycosis (Figure 1) and histoplasmosis [11] in non-HIV patients are rarely seen. Mycetoma, chromoblastomycosis, and sporotrichosis have not been reported from Malawi, although several cases of sporotrichosis are documented in neighbouring Zimbabwe [17]. Allergic and invasive fungal rhinosinusitis and fungal balls of the sinuses probably also occur but there are no data.

Tinea capitis is common, notably in school age children, with an estimated prevalence exceeding 670,900 (3780/100,000). This estimate relies on a point prevalence survey from Rwanda in which 21% of the children were found to have tinea capitis [18]. In a detailed study of children in south west Uganda, the most common causative organisms were: *Trichophyton violaceum* (the most common causative agent), followed by *Microsporum audouinii*, *T. soudanense*, and *T. rubrum* [19].

The prevalence of fungal keratitis is difficult to estimate, but cases are frequently seen in the eye department at Lions Sightfirst Eye hospital in Blantyre, Malawi. In one representative referral eye unit, one to two suspected cases of fungal keratitis are seen on a daily basis. This translates to at least 365 suspected cases per year from one eye unit, and considering that there are five dedicated eye units where most of these cases would be referred to, approximately 1825 cases are seen per year. It should be noted that within all the five Ophthalmology referral units, the suspected cases are treated empirically with any available antifungals (either topical and systemic), based on clinical judgment, as there are currently no diagnostic laboratory services to culture and grow fungus. This compromise in both the diagnostic and treatment modalities means that most cases of fungal keratitis are not managed properly and end up with complications including corneal scarring and visual loss.

We therefore estimate that 1,338,523 people in Malawi (7.54%) are affected by serious fungal infections. The estimates are just that, estimates, and substantial additional work would be necessary to fully document the actual annual caseload and prevalence.

## 4. Conclusions

This is the first attempt to estimate the burden of serious fungal infections in Malawi. Our estimates show that most life-threatening fungal infections in Malawi affect people living with HIV (PLHIV). This may be because there are more data available on the HIV population in Malawi than other groups of patients with immunosuppressive conditions. The lack of appropriate and rapid diagnostic tests makes it impossible to routinely come up with an accurate diagnosis, which thus affects the treatment. As an example of this, for those presenting with suspected cryptococcal meningitis, lumbar puncture is routinely done, but due to the unavailability of Cerebrospinal fluid (CSF) cryptococcal antigen assays, the routine test is CSF India Ink. Even this does not often come back within 24 h. Suspected patients are started on antifungals while waiting for the laboratory results, and depending on the results, treatment is either continued or discontinued.

It is anticipated that our work will create a debate and initiate more robust data collection to inform policy and practice change. Engagement of supporting funding partners and policy makers like the World Health Organization is needed to ensure that laboratory services can offer minimum diagnostic tests, and that antifungals on the WHO essential medicines list are made available in Malawi. Together, these would ensure that disability and deaths associated with treatable fungal infections are reduced.

## Figures and Tables

**Table 1:** The total estimated burden of fungal infections is 1,3358,523.

Infection	Number of Infections per Underlying Disorder per Year					Total Burden	Rate/100 K
	None	HIV/AIDS	Respiratory	Cancer/Tx	ICU		
**Superficial**							
Oral candidiasis		216,000				216,000	1217
Oesophageal candidiasis		73,000				73,000	411
Recurrent *Candida* vaginitis (≥4x/year)	326,960					326,960	3684
Fungal keratitis	1825					1825	10.3
Tinea capitis	670,900					670,900	3780
**Allergic**							
Allergic bronchopulmonary aspergillosis (ABPA)			11,190			11,190	63.0
Severe asthma with fungal sensitization (SAFS)			14,771			14,771	83.2
**Invasive Infections**							
Cryptococcal meningitis		8200				8200	47.3
*Pneumocystis* pneumonia		10,800				10,800	61.8
Candideamia				600	150	750	5
Invasive aspergillosis		1080		106		1186	6.7
Mucormycosis				30		30	0.2
Chronic pulmonary aspergillosis			2911			2911	16.4
**Total Burden Estimated**	**999,685**	**309,080**	**28,872**	**736**	**150**	**1,338,523**	
